# Biosensors integrated within wearable devices for monitoring chronic wound status

**DOI:** 10.1063/5.0220516

**Published:** 2025-02-04

**Authors:** Sabine Szunerits, Rabah Boukherroub, Christoph Kleber, Wolfgang Knoll, Jhonny Yunda, José Rumipamba, Guido Torres, Sorin Melinte

**Affiliations:** 1 Univ. Lille, CNRS, Univ. Polytechnique Hauts-de-France, UMR 8520—IEMN, F-59000 Lille, France; 2 Laboratory for Life Sciences and Technology (LiST), Faculty of Medicine and Dentistry, Danube Private University, 3500 Krems, Austria; 3 Université catholique de Louvain, ICTEAM, 1348 Louvain-la-Neuve, Belgium

## Abstract

Slowly healing wounds significantly affect the life quality of patients in different ways, due to constant pain, unpleasant odor, reduced mobility up to social isolation, and personal frustration. While remote wound management has become more widely accepted since the COVID-19 pandemic, delayed treatment remains frequent and results in several wound healing related complications. As inappropriate management of notably diabetic foot ulcers is linked to a high risk of amputation, effective management of wounds in a patient-centered manner remains important to be implemented. The integration of diagnostic devices into wound bandages is under way, owing to advancements in materials science and nanofabrication strategies as well as innovation in communication technologies together with machine learning and data-driven assessment tools. Leveraging advanced analytical approaches around local pH, temperature, pressure, and wound biomarker sensing is expected to facilitate adequate wound treatment. The state-of-the-art of time-resolved monitoring of the wound status by quantifying key physiological parameters as well as wound biomarkers' concentration is presented herewith. A special focus will be given to smart bandages with on-demand delivery capabilities for improved wound management.

## I. INTRODUCTION: WOUND MANAGEMENT

A chronic wound is damage to the skin that does not progress through the typical phases of healing in the expected timeframe and usually persists for more than 12 weeks. The most common types of chronic wounds are venous ulcers (VLUs)^1^ that typically appear on the legs (gaiter zone) due to poor blood circulation and diabetic foot ulcers (DFUs) that develop on the feet (forefoot) of people with diabetes owing to nerve damage and poor blood circulation.^2^ DFU is a common side effect of diabetics. Hyperglycemia is known to be associated with an increase in thromboxane A2, a potent platelet activator and vasoconstrictor that can have pathological consequences when activation is uncontrolled.

**FIG. 1.**
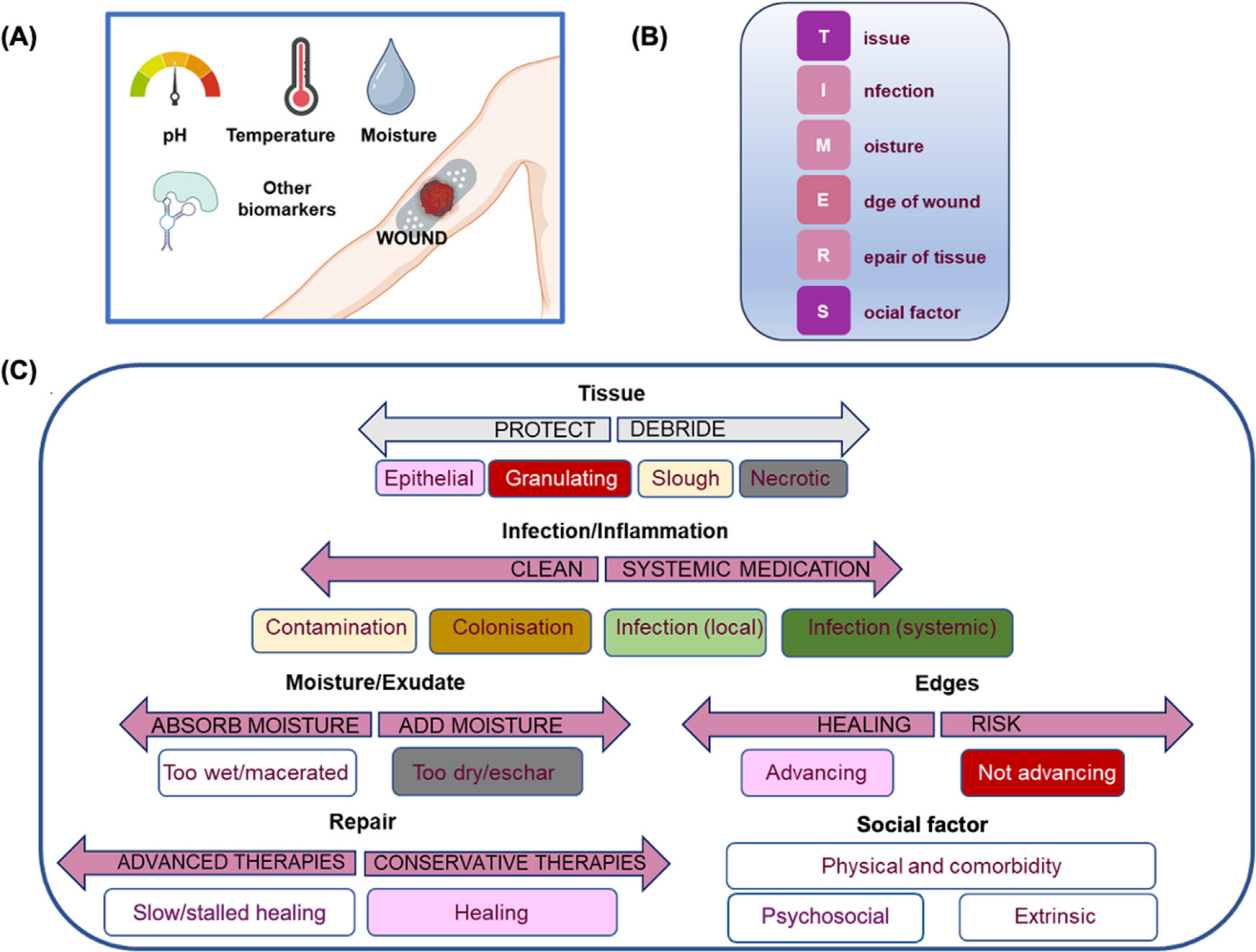
Wound care decisions can be made using the TIMERS concept. (A) pH, temperature, moisture, and biomarkers for continuous monitoring. (B) Elements of the TIMERS concept [created with BioRender software (BioRender.com)]. (C) Details of the TIMERS concept: boxes specify the state of the wound assessment and arrows indicate the action/treatment to be followed. Epithelium tissue appears as pink in the final stage of healing, red when healthy tissue is formed in the remodeling phase, brown due to devitalized tissue made of dead cells (slough), while necrotic tissue is black. Inflammation can persist due to infection requiring wound cleaning and treatment depending on the wound state. Moisture is essential to healing, and the treatment focuses on retaining and containing it within the wound bed. The measurement of the size and depth of the wound together with the identification of the edges is a key step in clinical assessment. Healing outcome and tissue repair depend on the chosen treatment approaches, based on expert experience. This Figure was constructed based on Refs. 3 and 4.

The evaluation of the wound state of DFU is currently guided by the qualitative “TIMERS” concept based on visual appearance and odor, parameters checked at every clinical visit (Fig. 1).^3–5^ The assessment necessitates experienced personnel and is based on medical staff judgment. It requires, in addition, frequent removal of the bandage, with the possibility to interrupt the wound healing process.

DFUs are associated with infections of the wound bed and the underlining reasons for limp amputations. These infections can spread to deeper tissues or the bloodstream and lead to sepsis, chronic pain and reduced mobility, which can significantly affect the quality of life. In severe cases, tissue necrosis and gangrene may necessitate amputation. Untreated chronic wounds also increase the risk of hospitalization, healthcare costs, and even death, especially in vulnerable populations. Therefore, timely and appropriate treatment is essential to prevent these severe consequences. This is, however, not possible without adequate early state diagnostics of ulcer infections. While classical symptoms of an infection are associated with pain, redness, and/or foot swelling, the implementation of wound sensors close to the wound bed that monitor pH, temperature, and wound biomarkers is expected to result in a better and earlier indication of the presence of infection.^2^ Although some current wound dressings exhibit antibacterial properties and accelerate wound closure,^3,4^ these bandages do not offer information about the physiological state of the wound environment.

An increased number of research teams sees wearable technology for wound care purpose as an important concept to be integrated into wound management.^5–12^ Wearables equipped for *in situ* monitoring of wound pH are probably one of the most advanced technologies.^13–16^ Wearable pH sensors, based on colorimetric detection, were one of the first approaches, as they can be easily implemented by embedding pH sensitive dyes within wound bandage material.^16^ These bandages are based on pH-responsive hydrogel fibers loaded with brilliant yellow dye for wound pH monitoring in the range of 6–8. The change in pH could be visually recorded via a smart phone. The main drawback of this first generation of wound bandages with pH sensing capabilities is that the pH sensitive dye has limited pH range and often leaches into the wound.

This review is focused on the description of monitoring systems for the main parameters employed in chronic wound management (i.e., DFU and VLU) by clinicians in daily practice.^6–8^ Section II details the main advances in wound pH monitoring as an indicator of bacterial growth. Section III describes the data on temperature monitoring because it indicates the presence of infection. In Sec. IV, other physiological parameters and biomarkers (i.e., oxygen, hydration, uric acid, and protein biomarkers) that are helpful indicators in wound management are discussed. Finally, Sec. V addresses the current monitoring systems in wound management and future perspectives considering artificial intelligence and multimodal systems.

## II. pH MONITORING

**FIG. 2.**
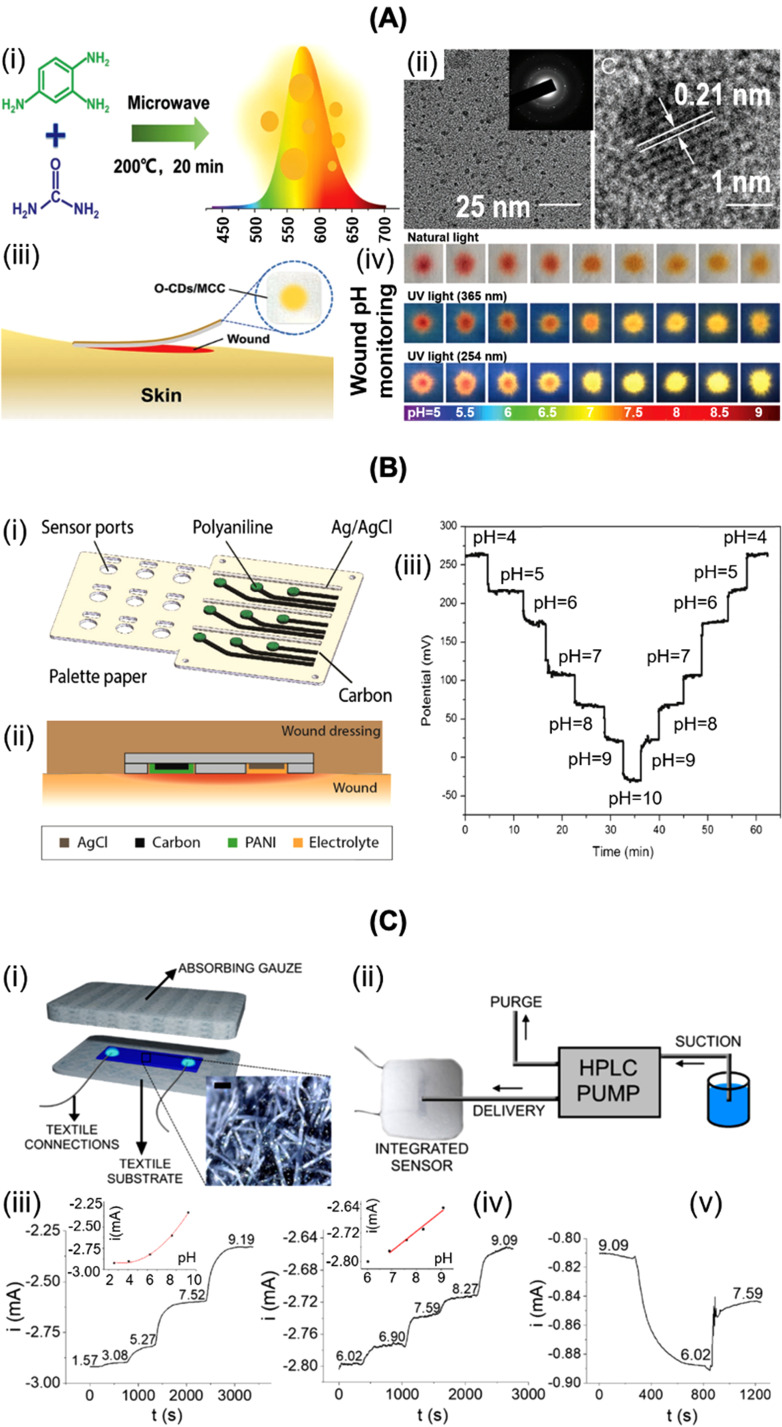
Advances in pH sensors for wounds. (A) (i) Synthesis of O-CDs. (ii) Transmission electron microscopy image and corresponding electron diffraction pattern of O-CDs. (iii) Conceptual view of the practical application. (iv) Change in color under white light irradiation as well as fluorescence images under natural, 365 and 254 nm excitations of O-CDs/MCC at various pH values. [Reprint with permission from Yang *et al.*, Small **15**, 1902823 (2019). Copyright 2019 John Wiley and Sons]. (B) (i) Schematics of the pH sensor array. (ii) Cross section of embedded wound sensor. (iii) Sensor response to pH changes. [Reprint with permission from Rahimi *et al.*, Sens. Actuators, B **229**, 609–617 (2016). Copyright 2016 Elsevier]. (C) (i) Schematics of textile PEDOT:PSS/IrO_x_ pH sensor. (ii) Wound bandage sensor design. (iii) Sensor time response in universal buffer solution; inset is the calibration plot. (iv) Response of the sensor in simulated wound exudate; inset is the calibration plot. (v) Signal recovery in simulated wound exudate. [Reprint with permission from Mariani *et al.*, ACS Sens. **6**, 2366–2377 (2021). Copyright 2021 Authors, licensed under a Creative Commons Attribution (CC BY) license].

Healthy skin is acidic with pH 4–6, owing to the presence of free fatty acids on the skin surface, essential for barrier homeostasis and optimal in limiting bacterial proliferation.^16,17^ Chronic wounds are characterized, however, by a more alkaline pH resulting from the loss of carbon dioxide (CO_2_) from the wound bed and the presence of urease-producing organisms, generating ammonia.^18–21^ In a recent study by some of us, a pH between 7.0 and 8.5 was indeed determined in wound fluids from patients with chronic diabetic foot ulcers;^22^ other reports indicated even a pH increase to 10.^23^ Such a large difference in pH argues that monitoring of pH levels in wounds represents a promising strategy. Carbon quantum dots (O-CDs) emitting orange light, rather than pH sensitive dyes, were proposed by Yang *et al.* as a pH indicator.^24^ When assembled in a medical cotton cloth (MCC), color changes from orange to yellow related to pH variation from 5 to 9 were observed [Fig. 2(a)]. An electrochemical pH sensor was proposed, among others, by Rahimi *et al.*^21^ and was based on the pH sensitivity of polyaniline (PANI) [Fig. 2(b)]. The emeraldine form of PANI was protonated to its emeraldine salt, under acidic pH, with a subsequent change in conductivity. This process is reversed under alkaline conditions.^9,21,25,26^ Typically, PANI sensors operate in a pH range between 4 and 10 with a potentiometric 50 mV/pH or capacitive 30 nF/pH sensitivity. Mariani *et al.*^27^ recently described [Fig. 2(c)] the potential of poly(3,4-ethylenedioxythiophene):polystyrene sulfonate/iridium oxide (PEDOT:PSS/IrO_x_) particles encased in a wound dressing structure for pH sensing based on potentiometric transduction. This sensor recorded a sensitivity of 59 *μ*A/pH in the pH range 6–9. A microneedle potentiometric sensor for pH transdermal measurements was lately reported by García-Guzmán *et al.*^28^ This sharp stainless-steel microneedle pH sensor, modified with carbon nanotubes and Ag/AgCl coatings acting as working and reference electrodes, respectively, showed the capacity of continuous pH monitoring in *in vivo* models. Its translation to wound pH measurements is pending.

Despite the significant progress in wound bandages, the practical application of integrated pH sensors for real-time pH monitoring still needs to overcome a series of bottlenecks. Due to small wound pH changes at early wound infections, the sensitivity of the sensors poses major challenges. As pH sensors necessitate a liquid environment, the restricted accessibility of wound fluid limits their usefulness and entangles engineering challenges. While integration of electrical detection-based pH sensors is constrained by power and calibration issues, the development of wound dressings capable of *in situ* analysis of wound markers gained attraction in recent years.^9,29–32^ To be noted is that strategies for the diagnosis as well as treatment of wound infections remain a great challenge due to the occurrence of biofilm formation, delayed healing, and drug resistance. Intravenous injection and oral administration of antibiotics are generally used for acute wound infections and are complemented in chronic wound infections with antimicrobial cream gels to eliminate deeper infection caused by the migration of bacteria or fungi to the subcutaneous tissues.^30^ Many antimicrobial wound dressings have been designed and comprised colloidal silver or cadexomer iodine as antimicrobials. A multifunctional dressing (GelDerm) has been proposed for a combined diagnostic/healing approach by the team of Akbari in 2017.^31^ It is based on monitoring the wounds according to the pH level and a wireless interface for quantification of the wound condition. Additionally, this study demonstrated the ability of GelDerm to eradicate bacteria by the sustained release of antibiotics such as gentamicin. A topical delivery of antibiotics minimizes limitations of intravenous drug administration with the advantage of a local high dose administration to the wound site. Certainly, battery-less implementation of dressing, as proposed by Xu *et al.*,^32^ further complemented by body energy harvesting wearables, represents a promising avenue for smart bandages (Table I). The team proposed a theragnostic closed-loop approach for the diagnostics and treatment of an infected wound. Temperature, pH, and uric acid of the wound were detected by the sensing part of the smart wound bandage to assess wound conditions, while antibiotics could be delivered via an electrically controlled delivery mode.^32^ Similar considerations apply to anti-fungal treatment of wounds^33^ using *Candida albicans* and filamentous fungi often present in chronic nonhealing wounds. There is currently no closed-loop device reported for simultaneous wound sensing and delivery of anti-fungal agents such as azoles, polyenes, and echinocandins.

**TABLE I.** Synopsis of current implementation of on-demand therapy bandages with wireless, continuous wound status monitoring.

Diagnostic	Drug delivery	Technology to release drug	Communication protocol	Reference
pH	Gentamicin	Passive	Bluetooth	31
pH Temperature Uric acid	Cefazolin	Electrical	Near-field communication	32
Temperature Moisture	Electrical stimulation	Electrical	Near-field communication	42
Temperature	Insulin Ramipril	Passive / Electrothermal / Photothermal	Bluetooth	43 44
Temperature	Ampicillin	Electrothermal	Near-field communication	45
Temperature	Parathyroid hormone, dextran, and doxorubicin	Electrothermal	Near-field communication	46

## III. TEMPERATURE MONITORING

Temperature changes are common indicators for assessing the wound status. The temperature in the wound bed and its vicinity is typically > 37.8 °C, with an increase in wound temperature >2.2 °C being an early sign of inflammation and/or infection. A decrease in temperature on the other hand indicates wound vasoconstriction.

**FIG. 3.**
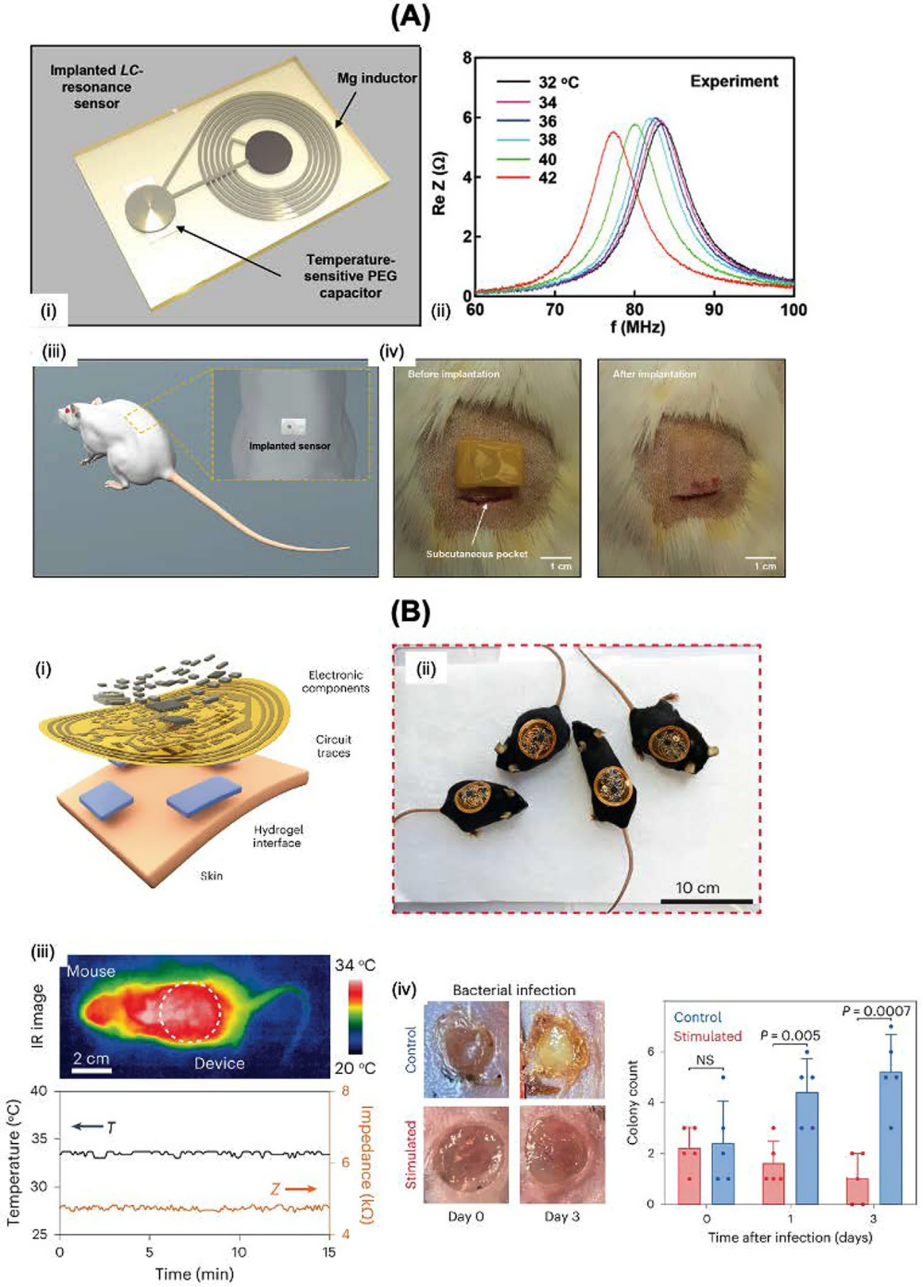
Temperature sensing related to wounds. (A) (i) Design of the temperature sensors based on a spiral coil inductor (*L*) and PEG-based capacitor (*C*). (ii) Shift of the *LC*-resonance peak *vs.* temperature. (iii) *In vivo* subcutaneous temperature measurements were taken with sensors implanted in rats. (iv) Details of the implantation procedure. [Reprint with permission from Lu *et al.*, Adv. Healthcare Mater. **9**, 2000942 (2020). Copyright 2020 John Wiley and Sons]. (B) (i) Flexible circuit board and conducting adhesive hydrogel integrated onto the bandage. (ii) Photographs of mice wearing the wound bandage. (iii) Infrared image of a mouse wearing the smart bandage (top) and traces of wirelessly sensed temperature and impedance (bottom). (iv) Photographs and quantitative comparison of wounds infected with *E. coli*, with and without stimulation. [Reprint with permission from Jiang *et al.*, Nat. Biotechnol. **41**, 652–662 (2023). Copyright 2023 Springer Nature].

The temperature of the wound bed is easily measured via infrared camera systems or resistance-based temperature devices.^34^ Infrared sensors integrated in smartphones are commonly used to generate high-resolution 2D thermal images to allow adequate wound treatment. Yet, the removal of the wound dressings is required for the assessment of the temperature, with the possibility of secondary damage to the wound. The use of thermochromic sensors based on encapsulating phase change materials within flexible materials, such as polyvinyl alcohol/polyurethane composite membranes, is one way to overcome this issue.^35^ Such sensors exhibit a resolution of about 0.2 °C and a response time of 1–50 s. Electrochemical means for wound temperature detection dates back to the work by Kim *et al.*^36^ where platinum sensors were incorporated into polydimethylsiloxane. Since this seminal work, a large variety of epidermal temperature sensors that map the temperature distribution in the wound area has been reported.^37–39^ Electronic tattoos, placed on human skin for noninvasive sensing, have attracted much interest. A self-healing electronic tattoo based on printing a graphene/silk fibroin/Ca^2+^ suspension onto skin was described by Wang *et al.*^39^ The graphene flakes induce responsiveness to strain, moisture, and temperature variations, representing a promising material for epidermal electronics. A bioresorbable, wireless, and power-free resonance-based temperature sensor [Fig. 3(a)] was designed by Lu *et al.* in 2000.^40^ The sensor consisted of polyethylene glycol (PEG), a temperature dependent dielectric, a magnesium foil inductor, and poly-lactic acid capacitors.

A precision of <0.05 °C was reached and the dressing showed the capability to monitor infection in internal wounds with high accuracy. However, the degradation rate of the sensor is fast accompanied by a considerable drop in performance, limiting its clinical applications. The team of Deferme reported lately on the development of a stretchable bandage for the concurrent sensing of temperature and strain; the device was further validated *in vivo*.^41^ The sensors were prepared on thermoplastic polyurethane foils, offering favorable features like stretchability, good transparency, and biocompatibility, by using PEDOT:PSS inks for sensor fabrication.

The state-of-the-art of smart bandage is the one described by Bao *et al.*^42^ It incorporates wound sensors and stimulators via a flexible bioelectric system consisting of wirelessly powered, close-loop sensing and stimulating circuits with skin-interfacing hydrogel electrodes with on-demand adhesion and detachment properties [Fig. 3(b)]. The device can be applied for continuous monitoring of skin impedance and temperature and can deliver stimulation in response to the wound environment. Other dressings in development,^43–46^ based on temperature-sensitive drug reservoirs,^43,44,47,48^ with wireless temperature monitoring and biologics delivery capabilities, are summarized in Table I.

## IV. OTHER WOUND MARKERS

### A. Oxygen

**FIG. 4.**
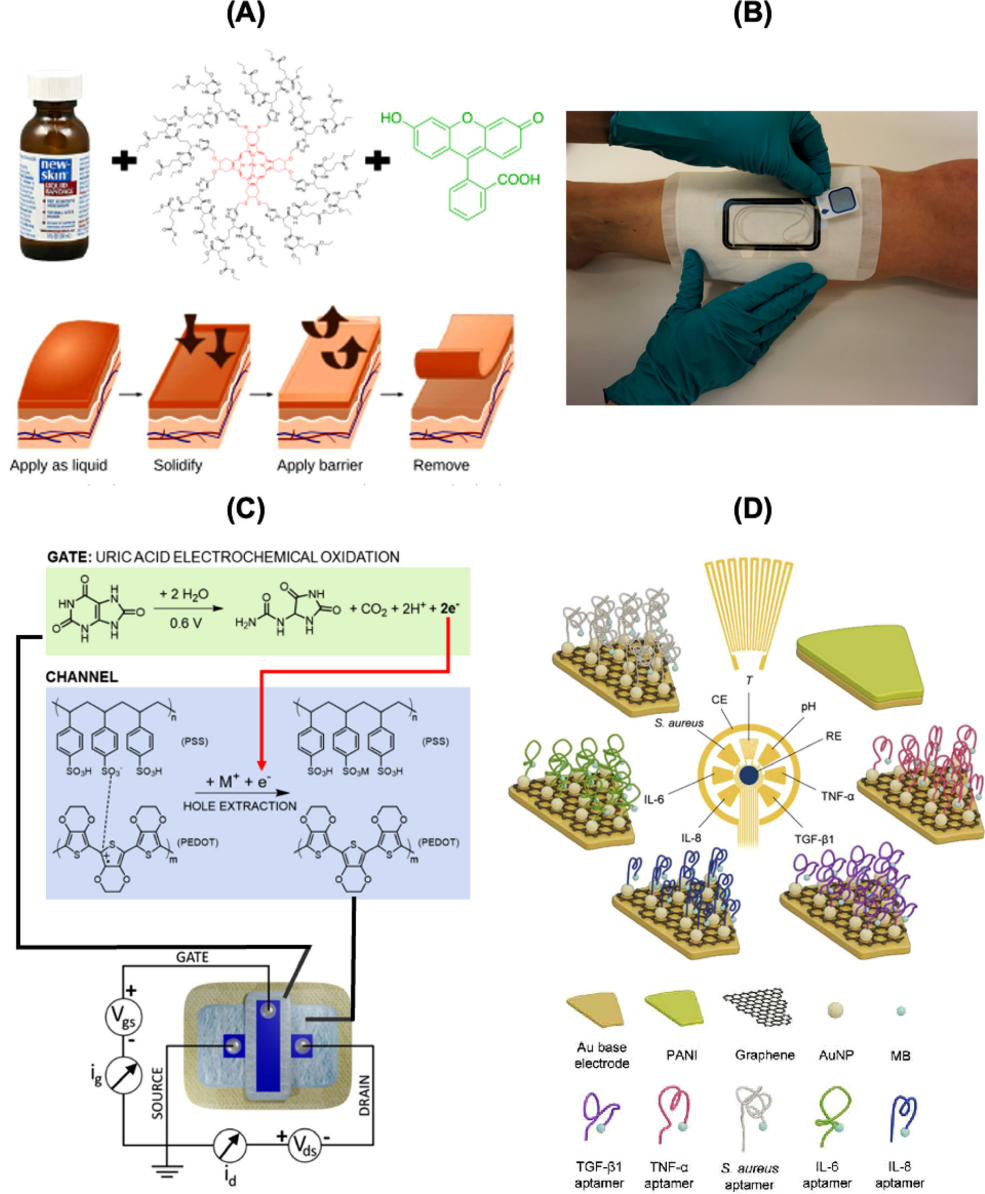
Detection of wound biomarkers. (A) Top: The various components of the liquid bandage formulation: New-Skin^®^ ethanol-nitrocellulose matrix, oxygen-sensing metalloporphyrin, and fluorescein dye. Bottom: Protocol for bandage fabrication. [Reprint with permission from Marks *et al.*, Sci. Adv. **6**, eabd1061 (2020). Copyright 2020 American Association for the Advancement of Science]. (B) Picture of Absorbest Fuktsensor on wound dressing. Image of the dressing with conductor coil and display placed outside. [Reprint with permission from Henricson *et al.*, Skin Res. Technol. **27**, 918–924 (2021). Copyright 2021 Authors, licensed under a Creative Commons Attribution (CC BY) license]. (C) OECT working mechanism for potentiostatic UA quantification. [Reprint with permission from Arcangeli *et al.*, ACS Sens. **8**, 1593–1608 (2023). Copyright 2023, American Chemical Society]. (D) Multianalyte skin sensor. [Reprint with permission from Gao *et al.*, Sci. Adv. **7**, eabg9614 (2021). Copyright 2021 Authors, licensed under a Creative Commons Attribution (CC BY) license].

Wound oxygenation is determinant for wound healing, and providing wound beds with sufficient oxygen enhances cell proliferation as well as angiogenesis. Oxygen also helps in producing reactive oxygen species (ROS) that protect wounds from infections and stimulate growth factors' release. The literature on wearable sensors for detecting tissue oxygenation within wound beds is vast with mainly optical sensors and electrochemical concepts being applied.^49^ Electrochemical sensors are based on the reduction of oxygen at a fixed potential where the measured current is proportional to dissolved oxygen levels. Interestingly, while such sensors have been widely used, most of them lack flexibility and cannot be integrated with the human body. One example of a skin-adapted oxygen monitoring sensor is that proposed by Ashley *et al.*^50^ The technology is under improvement as the low limit of the linear range is above 8 mg/L (corresponding to 53 [O_2_]% in air-saturated biological fluids), restricting its use for wound monitoring, where oxygen concentrations vary between 0 and 28 [O_2_]%. A phosphorescence-based, wireless tissue oximetry method was proposed by Marks *et al.*^51^ It uses the phosphorescence of metalloporphyrin [Fig. 4(a)] integrated as paintable formulation with the dressing to map tissue oxygenation.

### B. Hydration

Moisture sensing in wound beds is typically monitored through impedimetric or capacitive measurements using electrodes (e.g., PEDOT:PSS) deposited onto medical dressings.^52^ Acknowledging that a wet environment will trigger maceration, while a dry environment will impede healing, moisture sensors can facilitate the management of exudative wounds. McColl *et al.*^53^ introduced an array of 2 × 4 sensors placed between the wound and the dressing interface, mapping a wound surface of 6 × 6 cm^2^. The design relies on Ag/AgCl electrodes and was validated by *in vitro* tests for a high salt containing solution that is pumped at a rate of 0.5 ml/cm^2^ for 24 h, equivalent to a moderately exuding wound. The moisture level is read on a straightforward 1–5 scale, where 1 means very dry (>200 kΩ), 3 (15–50 kΩ) indicates the ideal moisture level necessary for healing, and 5 means very wet (<1.4 kΩ). Milne *et al.*^54^ used the same sensor, which by that time had already been marketed as WoundSense (Ohmedics Ltd, UK), in *in vivo* studies for real-time wound moisture monitoring. The moisture sensor provided primary indication of when the dressing should be changed (scale 5).

Henricson *et al.*^55^ used the commercial DryMax Extra Soft bandage (Absorbest AB, Sweden) together with the Absorbest Fuktsensor (Absorbest AB, Sweden), a non-sterile medical device based on carbon-Zn/MnO_2_ electrodes, an Ag coiled conductor coupled to an electrochromic display [Fig. 4(b)]. The operating principle is based on ions found in the wound exudate, which in contact with the electrodes generate a small current that activates the display. The dressing is suitable for highly exudative wounds (electrical impedance <1.4 kΩ), it runs without batteries or software and it was validated in an *in vivo* pilot study with five patients.

### C. Uric acid

Uric acid (UA) in wound exudate is present at concentrations of 220–750 *μ*M, while UA levels <220 *μ*M are associated with infection.^56^ The decrease in UA concentration is due to the capability of bacteria to metabolize UA to 5-hydroxyisourate. An increased level of UA points to ongoing necrosis of the tissue as the dead cells release adenosine triphosphate, which degrades to UA.^57^ The first smart bandages integrating UA detection were based on the use of enzymes to sense UA.^58–60^ Lately, PEDOT:PSS ink was applied to develop an organic electrochemical transistor (OECT) device for UA sensing in wound exudate [Fig. 4(c)]. The device sensed UA concentration changes, under flow conditions, in the biologically relevant concentration range, paving the way for *in situ* wound management.^61^

### D. Protein biomarkers

Some of us have validated the use of a matrix metalloproteinase (MMP) sensing field effect transistor as an alternative for monitoring wound closure. MMP-9 is upregulated in wounds with levels of 1.5–912 pM, whereas MMP-9 levels in healthy persons are <1.5 pM and could be detected by a graphene-based field effect transistor using aptamers as bioreceptors.^22^

Sensors targeting inflammation cytokines, such as Il-6, Il-8, TNF-α, and TGF-β1, are another way for obtaining information about the inflammation state of the wound. Aptamer-modified gold electrodes, further functionalized with Au nanoparticles (AuNPs) and graphene, were integrated into dressings comprising microfluidic exudate collection.^62^ This is one of the limited examples of a multiplexed wound sensor that integrates temperature and pH reading (via PANI transducer) as well as the detection of *Staphylococcus aureus* [Fig. 4(d)].^62^ The sensing principle relies on hairpin aptamer structures modified with methylene blue (MB). The aptamer structure experiences a conformational change during target binding with MB moving away from the electrodes, resulting in a decrease in the recorded redox current. This system was robust and could be applied once a week for five consecutive weeks to assess wound exudates from nonhealing wounds.

The formation of bacterial biofilms is a challenging task, leading to chronic wounds. As bacterial aggregates are typically <100 *μ*m in size, they cannot be identified by the naked eye. Fortunately, bacteria express virulence factors (e.g., rhamnolipids from *Pseudomonas aeruginosa*), which can be used for sensing the presence of bacterial colonies. The team of Jenkins^63^ proposed a sensor comprising liposomes loaded with self-quenching fluorescent dyes. The sensor was able to detect virulence factors that bacteria express in the early stages of biofilm formation.

## V. DISCUSSION AND PERSPECTIVES

When monitoring DFUs and VLUs, a set of key parameters, such as temperature, pH, hydration, and specific inflammatory biomarkers, like cytokines (i.e., IL-6 and TNF-α) and proteases (i.e., MMPs), can be used to assess wound status, detect early signs of infection, and guide treatment strategies. However, differences in the underlying causes of DFU and VLU require different follow-up approaches. For example, DFU presents a more significant challenge in wound management due to the metabolic complications of diabetes, so there is also a need to develop biosensors to monitor glucose levels. Meanwhile, VLU focuses more on factors related to venous insufficiency, requiring the monitoring of cytokines such as vascular endothelial growth factor, as well as increased fluid leakage from blood vessels to surrounding tissues,^64^ resulting in a greater volume of wound exudate.

The design and manufacture of biosensors present many challenges, since employed materials must demonstrate unique mechanical (i.e., flexibility, softness, permeability, and elasticity) and biochemical (i.e., biocompatibility and biodegradability) properties that allow for comfort and functionality in treating chronic wounds.^65–69^ Flexible substrates allow the integrated sensors to adapt to irregular wound surfaces without causing discomfort. For example, materials such as silicone elastomers, polyurethanes (e.g., Tegaderm^TM^), natural polymers (e.g., cellulose and chitosan), and synthetic polymers (e.g., polyvinylacetate, polydimethylsiloxane, and polyethylene glycol) are used as fabrication supports. Functional layers include, for example, biocompatible hydrogels that have been developed for exudate absorption. Moreover, these hydrogels can be loaded with antioxidants to achieve the antioxidant effect needed in the healing process.^70^ Gelatin methacrylate hydrogels encapsulated with ascorbyl phosphate have shown to accelerate full-thickness wound repair in diabetic wounds through multiple biological pathways, including reactive oxygen species scavenging, angiogenesis, neurogenesis, and collagen remodeling.^71^ Fat emulsion with methacrylate hyaluronic acid hybrid hydrogel has reduced IL-1/β production at the wound site, improved blood vessel density, and enhanced diabetic wound healing rate and tissue epithelialization.^72^ Furthermore, the use of nanomaterials such as metal–organic frameworks (integrating, e.g., copper and zinc) in combination with natural polymers (e.g., chitosan) contributed to the healing process and had antimicrobial properties.^73^

**FIG. 5.**
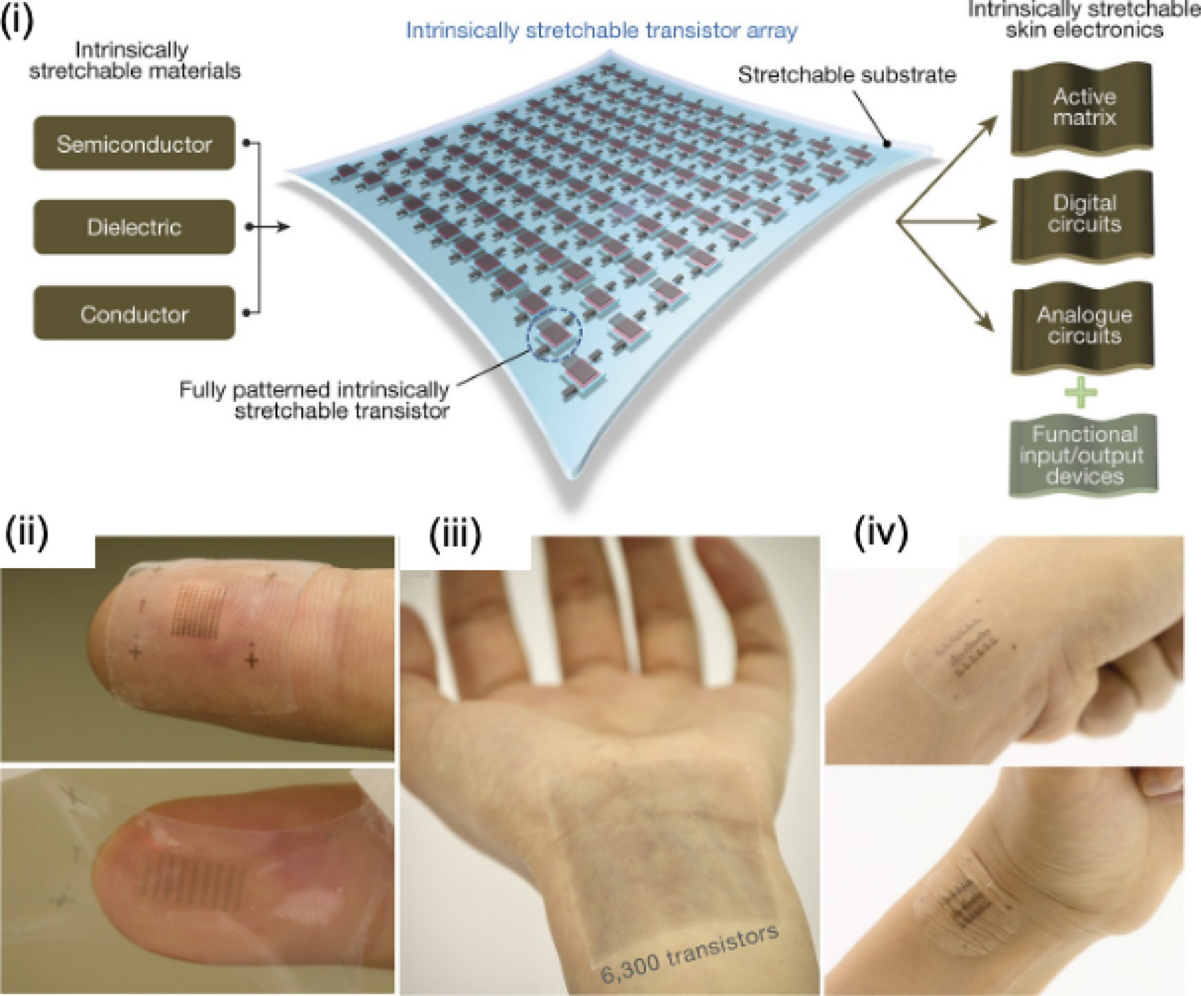
Stretchable skin electronics. Flexible and transparent electronics allow simultaneous monitoring of biomarkers and visual inspection. (i) Descriptive diagram of a transistor array manufactured from intrinsically stretchable and transparent materials. (ii) An array of 108 stretchable transistors attached conformably to a fingertip. (iii) An array of 6300 stretchable transistors attached conformably to an inner wrist. (iv) An array of stretchable transistors attached conformably to a bent wrist. [Reproduced with permission from Wang *et al.*, Nature **555**, 83–88 (2018). Copyright 2018 Nature].

In clinical practice, wounds are typically assessed using other methods than biosensors, such as visual assessment. The main advantage of wearable devices is that they allow for noninvasive continuous monitoring, but they cannot wholly replace the traditional visual method. Typically, wearable devices are placed on the wound and must be removed for visual inspection, but this entails extra work and more resources. Therefore, new approaches in the field focus on fabricating sensors using transparent materials that are optically and mechanically imperceptive, allowing simultaneous biomarker monitoring and visual inspection without the need to remove the device.^74^ This is a considerable challenge because the sensors are manufactured employing traditional conductive metals, mainly used in the transducers and electrical interconnections. To fulfill this requirement, ultra-thin metal films have been used to make integrated sensors imperceptible to the naked eye, exploiting conductive networks in the form of nanoparticles or nanowires. Carbon-based nanomaterials such as graphene and its derivatives (reduced graphene oxide) in single- or multi-layer films and carbon nanotubes are increasingly being used, owing to their high conductivity, mechanical, and transparency properties. An example of such devices can be seen in Fig. 5, highlighting a transistor array manufactured from intrinsically stretchable and transparent materials.^75^

Finally, wearable devices produce a lot of continuous information (i.e., time series), which is generally difficult to interpret, so the use of artificial intelligence (AI) (i.e., machine learning and deep learning) has revolutionized the field by allowing accurate physiological data monitoring and interpretation. In the case of chronic wound management, sensor data have the potential to be used to classify wound healing status using physiological parameters (e.g., pH, temperature, and oxygen level) and biomarkers (e.g., uric acid, glucose level) as well as electrical bioimpedance of the skin and to predict healing time. Algorithms based on traditional machine learning [i.e., artificial neural networks (ANN), support vector machines (SVMs), and gradient boosting machines (GBMs)] and deep learning [i.e., long short-term memory (LSTM)] have shown better performance with sensor data.^76,77^

**FIG. 6.**
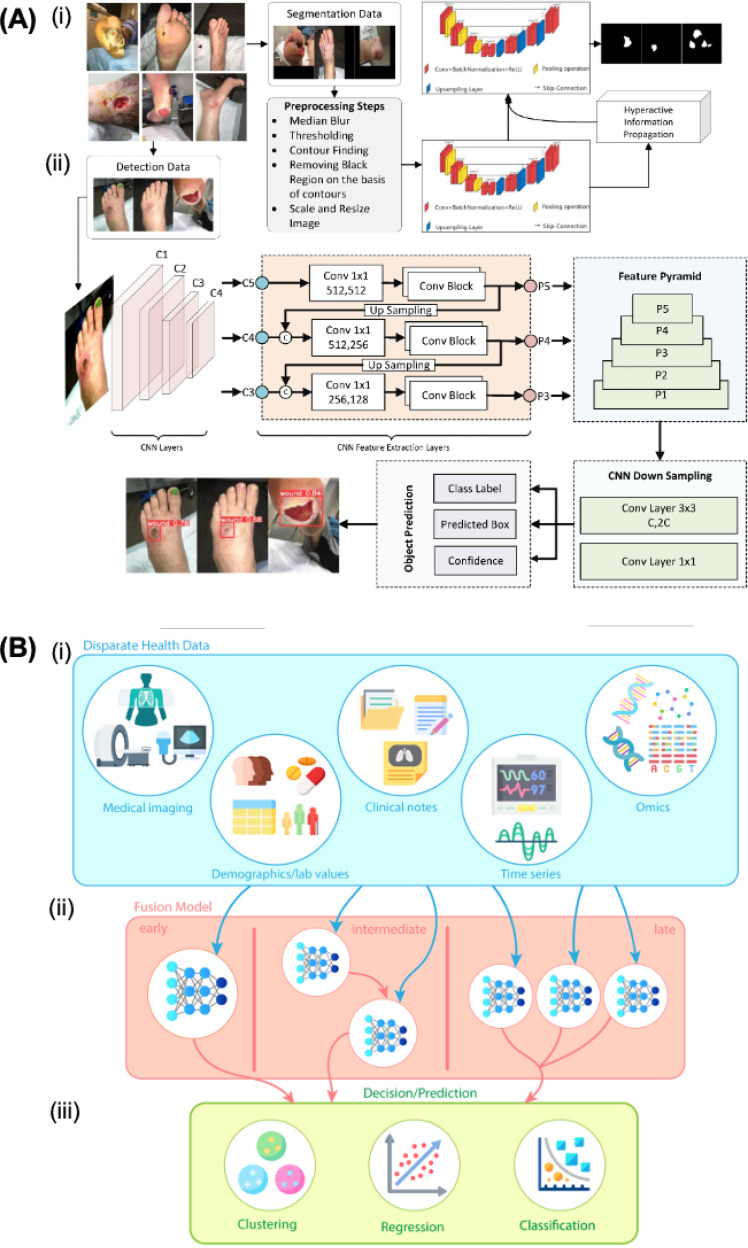
Artificial intelligence in wound management. (A) Artificial intelligence architectures in image-based wound management. (i) Wound segmentation based on Dual-Phase Hyperactive UNet. (ii) Wound detection based on YOLOv8. [Reprint with permission of Shah *et al.*, Healthcare **11**, 2840 (2023). Copyright 2023 Authors, licensed under a Creative Commons Attribution (CC BY) license]. (B) Multimodal artificial intelligence architecture for the management of chronic wounds employing disparate health databases (i), different types of data fusion models (ii), and decision/prediction multimodal algorithms (iii). [Reprint with permission of Kline *et al.*, npj Digital Med. **5**, 171 (2022). Copyright 2022 Authors, licensed under a Creative Commons Attribution (CC BY) license].

Unfortunately, current applications of AI in the field of wound management using smart sensors are scarce, because most of the work is focused on image interpretation (i.e., RGB, thermal and multispectral/hyperspectral images).^78,79^ One of the most common applications is wound type classification employing convolutional neural network (CNN)-based algorithms and their variants (i.e., AlexNet, VGG, ResNet, and YOLO). Another application is image segmentation, which allows locating the wound in the image automatically; among the most used networks are UNet. For example, in Fig. 6(a), a hybrid architecture that allows simultaneous detection (classification) and segmentation (location) of wounds can be observed.^80^ In this case, the segmentation part is based on a modification of UNet called Dual-Phase Hyperactive UNet, and the detection part is based on YOLOv8. New approaches tend to use multimodal AI systems, which allow handling data from different sources [i.e., biosensors, images, electronic health records (EHR)] and integrating them into a single algorithm, which helps assisting wound management in a fast, easy, and accurate way.^14,80,81^ An example is displayed in Fig. 6(b). Different data sources are available in clinical settings and AI helps with fusion models that allow the best features of each type of data to be exploited optimally in wound management.

## VI. CONCLUSIONS

Overall, the implementation of pH sensors in wound management has yielded promising results. The shortcomings of electrical-based pH sensors mainly relate to biocompatibility issues of the sensing layer and complex re-calibration procedures for long-term monitoring. The limitations of optical pH sensors include, next to the difficulty in data quantification and transmission, their low durability compared to electrical sensors and increased engineering complexity when on-demand feedback therapeutic approaches are to be implemented. In view of these hurdles, further efforts also should be devoted to pH monitoring. Skin temperature recording is highly useful for predicting the healing progression of wounds, and sensor arrays will likely become the dominant technology for variable, large-scale wounds^65,66^ by taking advantage of novel packaging methods for soft electronics.^67^ The monitoring of wound biomarkers is increasingly important as the understanding of metabolic pathway alterations in wounds is progressing together with computational modeling.^82^ The question remaining is whether the current transformative technologies for diagnostics, namely, battery-free wearables and artificial intelligence, could significantly complement the multimodal assessment of wound healing through time-resolved recording of specific parameters and edge computing.^68,69^ Such methodologies include feature extraction (e.g., principal components analysis), supervised (e.g., convolutional neural networks), unsupervised (e.g., Gaussian mixture models), or deep learning (e.g., convolutional neural networks and long short-term memory units) techniques to integrate and correlate different datasets of sensed parameters, expanding the overall analytical capabilities of wound management.^14^

## Data Availability

Data sharing is not applicable to this article as no new data were created or analyzed in this study.
